# New Books

**Published:** 2006-09

A Climate of Injustice: Global Inequality, North–South Politics, and Climate Policy

J. Timmons Roberts, Bradley C. Parks

Cambridge, MA:MIT Press, 2006. 384 pp. ISBN: 0-262-18256-4, $65

Academic Scientists at Work

Jeremy M. Boss, Susan H. Eckert

New York:Springer, 2006. 289 pp. ISBN: 0-387-32176-4, $29.95

Advanced Analysis of Gene Expression Microarray Data

Aidong Zhang

Hackensack, NJ:World Scientific Publishing Co., 2006. 356 pp. ISBN 981-256-645-7, $68

Atlas of Mammalian Chromosomes

Stephen J. O’Brien, Joan Menninger, William G. Nash

Hoboken, NJ:John Wiley & Sons, Inc., 2006. 760 pp. ISBN: 0-471-35015-X, $350

Bioinformatics For Dummies, 2nd ed.

Jean-Michel Claverie, Cedric Notredame

Hoboken, NJ:John Wiley & Sons, Inc., 2006. 432 pp. ISBN: 0-470-08985-7, $29.99

Climate Change, Justice and Future Generations

Edward A. Page

Burlington, VT:Ashgate Publishing Company, 2006. 224 pp. ISBN: 1-84376-184-X, $85.50

Energy and Culture: Perspectives on the Power to Work

Brendan Dooley

Burlington, VT:Ashgate Publishing Company, 2006. 264 pp. ISBN: 0-7546-4514-2, $114.95

Environmental Justice and Environmentalism: The Social Justice Challenge to the Environmental Movement

Ronald Sandler, Phaedra C. Pezzullo, eds.

Cambridge, MA:MIT Press, 2006. 352 pp. ISBN: 0-262-19552-6, $62

Evaluation of Certain Food Contaminants

World Health Organization

Geneva:World Health Organization Press, 2006. 107 pp. ISBN: 92-4-120930-5, $36

Implementing The Precautionary Principle: Perspectives and Prospects

Elizabeth Fisher, Judith Jones, eds.

Northhampton, MA:Edward Elgar Publishing Inc., 2006. 352 pp. ISBN: 1-84542-702-5, $121.50

Integrated Genomics: A Discovery-Based Laboratory Course

Guy Caldwell, Shelli Williams, Kim Caldwell

Hoboken, NJ:John Wiley & Sons, Inc., 2006. 240 pp. ISBN: 0-470-09501-6, $165

Pesticide Residues in Food—2004

World Health Organization

Geneva:World Health Organization Press, 2006. 723 pp. ISBN: 92-4-166520-3, $63

Phytoremediation of Metal-Contaminated Soils

Jean-Louis Morel, Guillaume Echevarria, Nadezhda Goncharova, eds.

New York:Springer, 2006 360 pp. ISBN: 1-4020-4686-3, $169

Preventing Disease through Healthy Environments: Towards an Estimate of the Environmental Burden of Disease

A. Prüss-Ustün, C. Corvalán

Geneva:World Health Organization Press, 2006. 104 pp. ISBN: 92-4-159382-2, $22.50

SARS: How a Global Epidemic was Stopped

World Health Organization

Geneva:World Health Organization Press, 2006, 317 pp. ISBN: 92-9061-213-4, $36

Smart Growth And Climate Change

Matthias Ruth, Roy F. Weston, eds.

Northhampton, MA:Edward Elgar Publishing Inc., 2006. 432 pp. ISBN: 1-84542-509-X, $104

Tetrachloroethene

World Health Organization

Geneva:World Health Organization Press, 2006. 120 pp. ISBN: 92-4-153068-5, $45

The International Yearbook Of Environmental and Resource Economics 2006/2007

Tom Tietenberg, Henk Folme, eds.

Northhampton, MA:Edward Elgar Publishing Inc., 2006. 320 pp. ISBN: 1-84542-723-8, $121.50

The Practical Bioinformatician

Limsoon Wong, ed.

Hackensack, NJ:World Scientific Publishing Co., 2006. 540 pp. ISBN 981-238-846-X, $100

Toxicants in Terrestrial Ecosystems: A Guide for the Analytical and Environmental Chemist

T.R. Crompton

New York:Springer, 2006. 268 pp, ISBN: 3-540-33694-X, $169

